# Nomophobia and mental health: are cell phones taking our sleep away?

**DOI:** 10.1192/j.eurpsy.2025.1437

**Published:** 2025-08-26

**Authors:** A. C. Matias-Martins, T. Vieira, A. Ferreira Silva, R. Machado Lopes, C. Almeida Rodrigues, F. Marinho Santos, P. Fonseca Coelho

**Affiliations:** 1 Psychiatry and Mental Health Department, Unidade Local de Saúde do Médio Tejo, Tomar, Portugal

## Abstract

**Introduction:**

The widespread use of mobile devices, particularly among young people, extends beyond entertainment to education and professional purposes. However, excessive smartphone use has led to health issues such as headaches, poor concentration, sleep problems, and anxiety. A condition called nomophobia, or the fear of being without a mobile phone, has emerged, though it’s not officially recognized as a psychiatric disorder. In adults, 20% experience mild nomophobia, 50% moderate, and 20% severe symptoms, which can be measured using the Nomophobia Questionnaire (NMP-Q). Smartphone overuse is also linked to psychopathological issues like insomnia and anxiety.

**Objectives:**

The aim of this study is to evaluate the impact of nomophobia in insomnia and anxiety.

**Methods:**

Non-systematic review of the literature regarding nomophobia and anxiety and insomnia. The research was carried out through the PubMed® database, using the terms “nomophobia”, “nomophobia and anxiety” and “nomophobia and insomnia”.

**Results:**

The included studies highlight that nomophobia is associated with higher anxiety levels in most individuals as well as a substantial correlation between nomophobia symptoms and insomnia.

**Conclusions:**

Nomophobia is increasing due to technological advancements and widespread access. Overuse of mobile phones is linked to psychopathologic symptoms, like anxiety and insomnia. Raising awareness and helping young adults manage their phone use is essential for promoting health and well-being as digital technologies become an integral part of daily life.

**Disclosure of Interest:**

None Declared

